# *COG5* variants lead to complex early onset retinal degeneration, upregulation of PERK and DNA damage

**DOI:** 10.1038/s41598-020-77394-3

**Published:** 2020-12-04

**Authors:** Sami Tabbarah, Erika Tavares, Jason Charish, Ajoy Vincent, Andrew Paterson, Matteo Di Scipio, Yue Yin, Roberto Mendoza-Londono, Jason Maynes, Elise Heon, Philippe P. Monnier

**Affiliations:** 1 Department of Ophthalmology and Vision Sciences, Faculty of Medicine, 340 College St., Toronto, M5T 3A9 Canada; 2 Program of Genetics and Genome Biology, The Peter Gilgan Centre for Research and Learning, 686 Bay St., Toronto, ON M5G 0A4 Canada; 3 Krembil Discovery Tower, Krembil Research Institute, Vision Division, KDT-8-417, 60 Leonard St, Toronto, ON M5T 2O8 Canada; 4 Department of Physiology, Faculty of Medicine, University of Toronto, 1 King’s College Circle, Toronto, ON M5S 1A8 Canada; 5 Department of Pediatrics, Division of Clinical Genetics, The Hospital for Sick Children, 555 University ave., Toronto, ON M5G 1X8 Canada; 6 Department of Anesthesia and Pain Medicine, The Hospital for Sick Children, 555 University ave., Toronto, ON M5G 1X8 Canada; 7 Program in Molecular Structure and Function, The Hospital for Sick Children, 555 University ave., Toronto, ON M5G 1X8 Canada

**Keywords:** Cell biology, Molecular biology, Neuroscience, Medical research

## Abstract

Leber congenital amaurosis (LCA), a form of autosomal recessive severe early-onset retinal degeneration, is an important cause of childhood blindness. This may be associated with systemic features or not. Here we identified *COG5* compound-heterozygous variants in patients affected with a complex LCA phenotype associated with microcephaly and skeletal dysplasia. COG5 is a component of the COG complex, which facilitates retrograde Golgi trafficking; if disrupted this can result in protein misfolding. To date, variants in *COG5* have been associated with a distinct congenital disorder of glycosylation (type IIi) and with a variant of Friedreich’s ataxia. We show that *COG5* variants can also result in fragmentation of the Golgi apparatus and upregulation of the UPR modulator, PKR-like endoplasmic reticulum kinase (PERK). In addition, upregulation of PERK induces DNA damage in cultured cells and in murine retina. This study identifies a novel role for COG5 in maintaining ER protein homeostasis and that disruption of that role results in activation of PERK and early-onset retinal degeneration, microcephaly and skeletal dysplasia. These results also highlight the importance of the UPR pathway in early-onset retinal dystrophy and as potential therapeutic targets for patients.

## Introduction

Severe retinal degeneration (RD) with early-onset and nystagmus is often referred to as Leber’s congenital amaurosis (LCA), which is a genetically heterogeneous form of inherited retinal disease (IRD)^1^ for which gene-specific treatments have been initiated^2^. LCA leads to severe dysfunction of rod and cone photoreceptors, low vision, nystagmus, and often results in total blindness. LCA may or may not be associated with systemic features (e.g. microcephaly, neurological features, etc.). To date, at least 25 genes are associated with LCA, which accounts for ~ 80% of cases. Given this genetic heterogeneity, there are numerous mechanisms underlying the resulting retinal degeneration, however these disease mechanisms typically do not involve glycosylation abnormalities^3^. Hence, glycosylation related genes have not been considered as LCA candidate genes.

COG5 is part of an eight-subunit Golgi-resident protein referred to as the conserved oligomeric golgi (COG) complex, which facilitates retrograde Golgi trafficking of vesicles that may include necessary glycosylation enzymes^4^. Variants in almost all the COG subunits have been linked to congenital disorders of glycosylation (CDGs), causing wide-ranging disabilities^5^. *COG5*-related disease (CGDIIi, MIM613612) is rare and is characterized by a range of anomalies that include mild to severe cognitive delay, motor dysfunction, microcephaly, hypotonia, short stature, liver disorders, and deafness^6^. More recently *COG5* variants have been associated with a Friedreich’s ataxia-like phenotype^7^. Though a few cases of uncharacterized blindness have been associated with a CGD phenotype, RD has not been associated with *COG5* variants^8^. There are no currently available treatments for any of the COG-CDGs.

Disruption in glycosylation or Golgi trafficking due to dysfunctional COG subunits could lead to an aggregation of misfolded proteins causing ER stress and activation of the unfolded protein response (UPR). The accumulation of unfolded proteins in the ER lumen activates three separate arms of the UPR in parallel but independently of one another^9^. The three UPR arms are referred to by the name of their initiating proteins or sensor domains: protein kinase RNA-like ER kinase (PERK), inositol-requiring protein 1α (IRE1α), and activating transcription factor 6 (ATF6)^10^. PERK is a transmembrane ER-protein with domains on both the luminal and cytosolic side of the ER and its kinase activity functions to phosphorylate the eukaryotic initiation factor-2α (eIF2α), which regulates transcription pathways to stop global protein translation^11,12^. The PERK-arm is also found to be part of the recruitment of DNA damage repair signalling of γ-H2A.X^13^.

Here we studied a non-consanguineous Filipino family of three affected individuals who presented with complex LCA associated with short stature, mild microcephaly and signs of skeletal dysplasia. No disease-causing variant was identified using clinical-base gene panel testing available through CLIA-approved laboratories. Through whole genome sequencing (GS) we identified two compound heterozygous variants in *COG5*, a gene not previously associated with LCA. We then demonstrate in vitro that expression of both *COG5* variants resulted in the activation of the PERK branch of the UPR with an increase in Golgi fragmentation, and induction of DNA damage.

## Results

### *COG5* variants are associated with severe retinal degeneration, microcephaly and skeletal dysplasia

**Figure 1:**
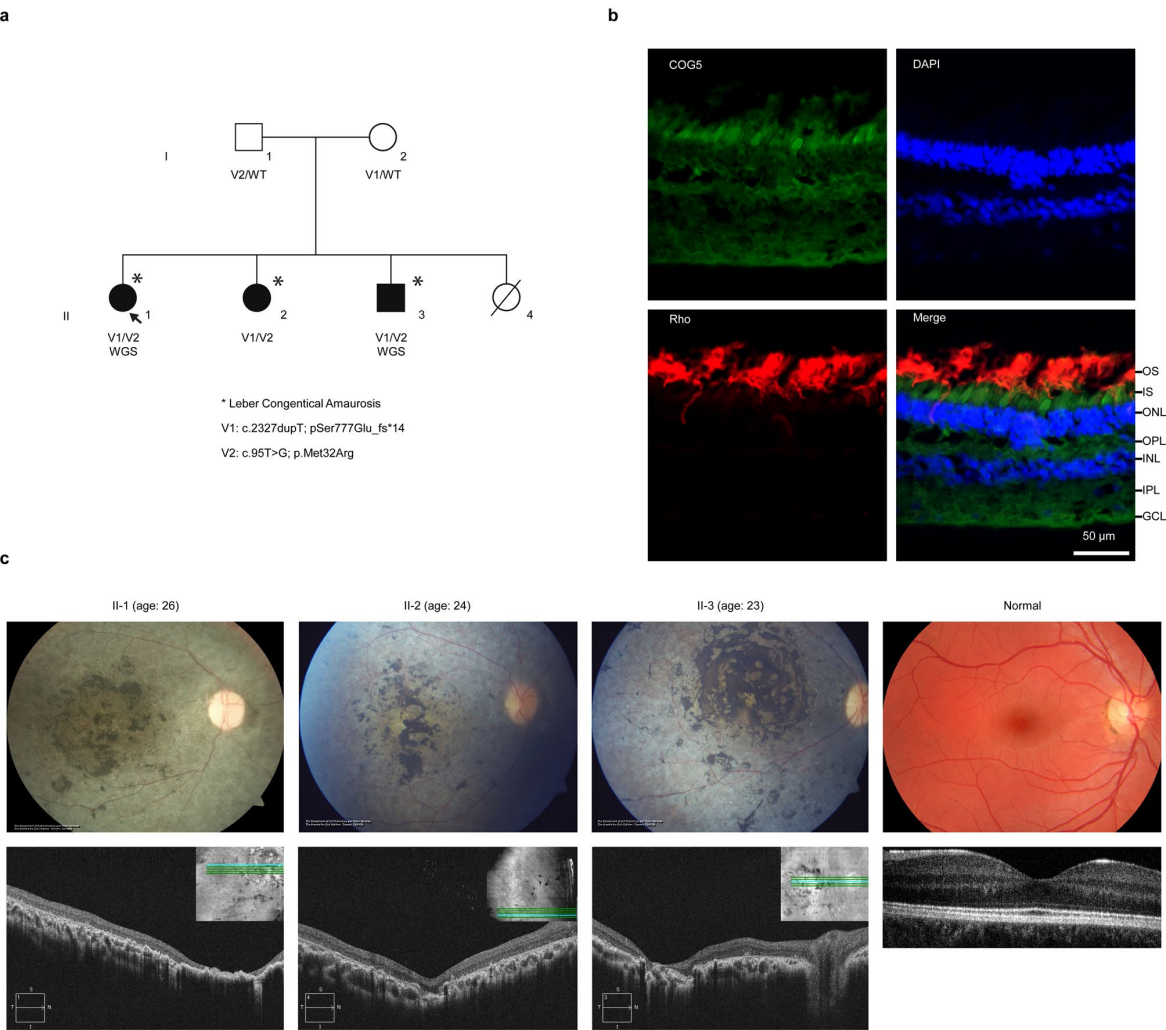
Details of family studied and retinal expression profile of COG5. (**a**) Pedigree chart represents the family of the proband (arrow) and her siblings, who all share a compound heterozygous variant in *COG5* and present with Leber’s congenital amaurosis (*) among other phenotypes. A fourth child was stillborn prior to the study. The parents have a heterozygous variant in *COG5* and do not present any phenotypes. [=]: wildtype. (**b**) COG5 retinal expression. Immunohistochemical staining performed on a sectioned retina from an adult human male showing COG5 strong staining in green in the inner segment. Rhodopsin (Rho) staining marks the outer segments (OS) of the photoreceptors. DAPI was used for nuclear staining; GCL = ganglion cell layer, IPL = inner plexiform layer, OPL = outer plexiform layer, ONL = outer nuclear layer, IS = inner segments layer of the photoreceptors, OS = outer segment of photoreceptor (**c**) Top: retinal images, Bottom: Optical coherence tomography (OCT) images with a top right corner insert indicating the region of the retina scanned. Far right images are of a normal retina with OCT below. Retinal imaging of the three cases was centered on the center of the retina (macula). Retinal degeneration is severe in all three cases with pigmentation, vessel attenuation, optic nerve pallor and thinning of the retina on OCT.

A non-consanguineous family of Filipino origin had 3 affected siblings (1 male, 2 females) with a complex form of Leber’s congenital amaurosis (LCA) (Fig. 1a). The asymptomatic parents were examined and were normal. The affection status of participants is summarized in Table 1. Briefly, all affected cases first presented with severe early-onset retinal degeneration (or LCA) with nystagmus, light aversion and legal blindness. All had a normal intellect, short stature, microcephaly and skeletal dysplasia. Cases II-1 and II-2 also had abnormal ossification of the skull which in case II-2 led to progressive combined sensory-neural and conductive hearing loss due to progressive oto-sclerosis. The vision loss was of early-onset as the three patients, now in their twenties, do not recall ever seeing better. At the most recent examination the patients were legally blind with a visual acuity of “counting fingers”. The ocular changes were limited to the retina, and typical of retinal degeneration (Fig. 1c). The cone ERGs were barely recordable (at ages 18–21 years) in all three cases, and the tracings were suggestive of severe cone-rod photoreceptor degeneration. The visual fields were severely constricted to the central ~ 5 degrees (Supplementary Fig. 1). In agreement with a role for COG5 in photoreceptor function, the protein was shown to be present in the inner segment of human photoreceptors where proteins are synthesized (Fig. 1b). In the peripheral human retina, the inner segments of cones are significantly larger than those of rods^14^, which can be used as a means to distinguish rods from cones on histological examinations^15^. We noted that within the same sections, cone photoreceptor inner segments often had a higher COG5 staining intensity compared to neighboring rod photoreceptor inner segments (Supplementary Fig. 2), which could support the cone-rod dystrophy phenotype observed.

**Table 1:** Clinical characteristics of patients studied with the identified *COG5* mutations.

Family subject	II-1	II-2	II-3
Gender	Female	Female	Male
**Systemic phenotype**			
Cognition	Normal	Normal	Normal
Motor function	Normal	Normal	Normal
Hearing ability	Normal	Bilateral sensorineural and conductive hearing loss	Normal
Gait	Normal	Unsteady	Normal
Stature	Short stature	Short stature	Short stature
	Skeletal dysplasia	Skeletal dysplasia	Skeletal dysplasia
	Scoliosis		
	Hip dysplasia		
Weight	45 kg	35 kg	36 kg
Height	138 cm	128 cm	133 cm
Dysmorphism	None	None	None
Head diameter	Microcephaly	Microcephaly	Microcephaly
	(< 4%ile, age 19)	(< 2%ile, age 18)	(< 3%ile, age 9)
Head and neck MRI	Skull base sclerosis	Skull thickening, progressive otosclerosis	Short corpus callosum
	Mild otosclerosis		
		T2 hypo-intensity in cerebellar dentate nuclei	
Clinical diagnosis	LCA	LCA	LCA
Visual acuity	Low (CF)	Low (CF)	Low(CF)
Nystagmus	Yes	Yes	Yes
Ant segm	Normal	Normal	Normal
Retinal condition	Diffuse mottling maculopathy	Diffuse mottling maculopathy	Diffuse mottling maculopathy
Visual field (diameter)	20–30° H × 10° V (IV4e) (21yrs)	40° central (III4e) (19yrs)	Peripheral island (IV4e) (18yrs)
ERG	Cone-rod dystrophy	Cone-rod dystrophy	Severe cone-rod dystrophy

LCA: Leber’s congenital amaurosis. Counting finger visual acuity is equivalent to 20/2000 at 2 feet. H: horizontal field diameter. V: vertical field diameter in degrees. IV4e and III4e refer to the size and intensity of the light stimulus used, IV4e is bigger than III4e. Ant Segm: anterior segment, CF: counting finger vision.

**Figure 2:**
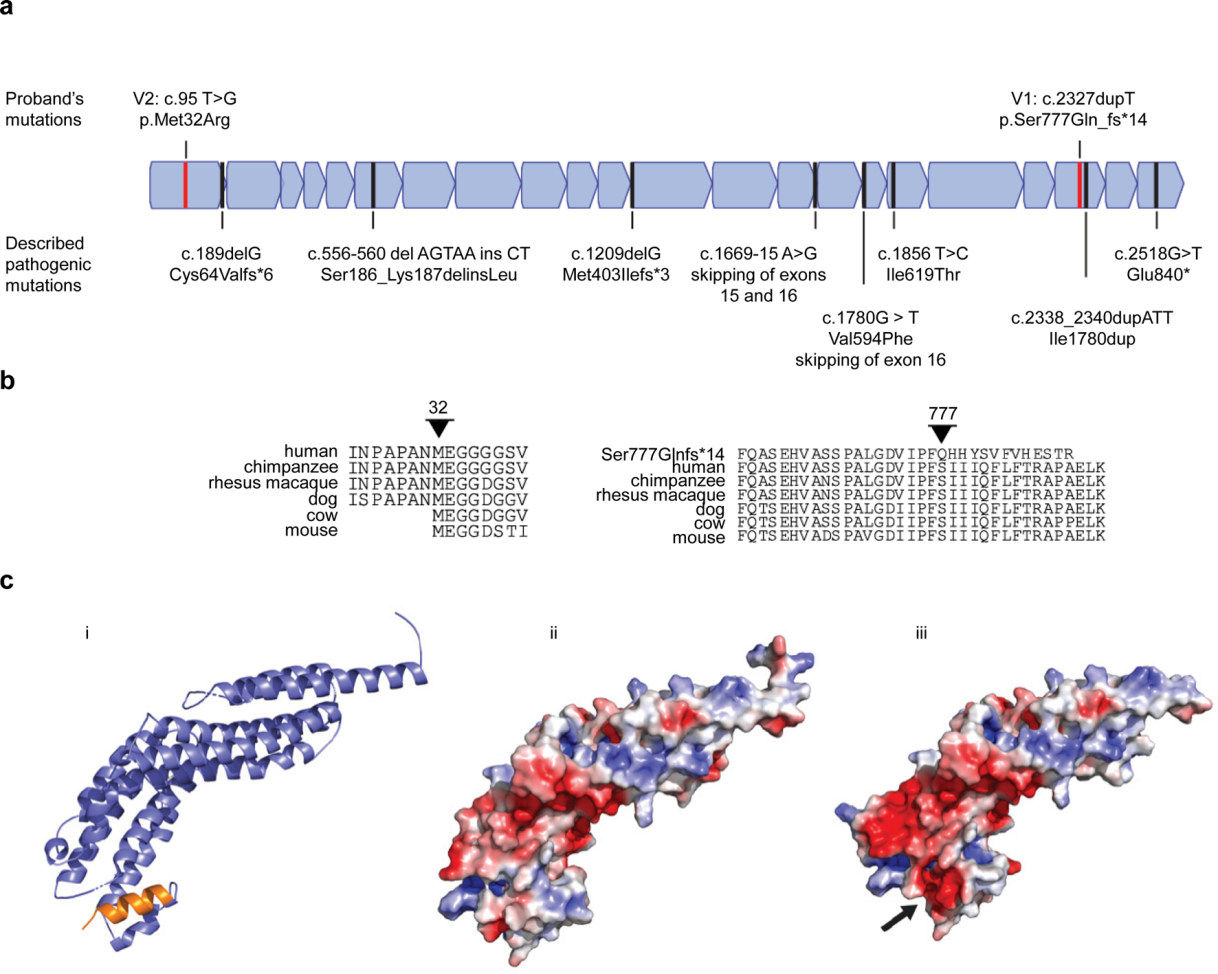
Retinal structural and functional phenotype of *COG5* patients. (**a**) Structural changes of the retina. (**a**) Schematic of *COG5* with location of the variants discussed (bold and above, red line). Previously identified variants in *COG5* are below except for Met32Arg in bold above as seen in this family. Exons with key domains are in blue. (**b**) Sequence homology showing conservation of the amino acids involved. (**c**) Structural Modeling of the COG5 c.2327 dupT variant (V1). (1) The C-terminal region of COG5 (residues 470–787) was modeled using the structure of sec6 (PDB ID 2FJI, model confidence score 98.3% with Phyre2, 11% sequence identity between sec6 and COG5 in this region). The frameshift resulting from the nucleotide duplication affects the amino acid composition of the extreme C-terminus of the structure, denoted in orange. The electrostatic surface potential of the wild-type (2) and frame-shifted (3) COG5 shows that the change in amino acids introduces a strongly negative charge into this C-terminal region (denoted by the arrow), dramatically affecting any protein: protein interactions in the COG supracomplex. For the electrostatic surface potential, white denotes electroneutral areas, red denotes electronegative areas and blue denotes electropositive areas. Electrostatic potential coloring is + / − 3.

Genetic analyses integrating the areas of maximum Lod score of 1.19 (chromosome 7q22) to our GS filtering pipeline identified *COG5* as the only potential candidate gene for which the variants segregated with the disease phenotype and was of functional relevance to the patient phenotype (Table S1). The two *COG5* variants identified were presumed pathogenic according to our internal protocols (Table S2 and Supplementary Fig. 3). Variant 1 was novel; Ser777Glnfs*14 (V1) while variant 2; Met32Arg (V2) was previously documented with another variant (Fig. 2a) in a patient affected with CDG without any ocular involvement^8^. V1 involved a residue conserved in mammals and resulted in a frameshift and a premature stop codon (Fig. 2b). Using the sequence from Uniprot (https://www.uniprot.org), we modeled the C-terminal half of the protein based on sec6ct2, which is an endo/exocytic protein^16^. The sequence alignment of V1 with the WT sequence of *COG5* falters at Phe778, which is located in the loop region of a helix bundle and, more specifically, a three-helix bundle that is a common structural motif. The sequence for the rest of the helix is SIIIQFLFT in the WT but here was changed to QHHYSVFV due to the frameshift variant. This likely breaks that final helix but also dramatically changes the charge status (Fig. 2c). This variant could affect the protein through two mechanisms: (1) alteration of its three-helix bundle, or (2) inducing a premature stop codon.

### *COG5* variants lead to ER stress and UPR activation

Reverse transcribed PCR of lymphoblasts-derived *COG5* RNA from the proband, mother, and control revealed both alleles were transcribed to RNA in the patient II-1. The allele carrying V1 (Ser777Glnfs*14) was partially expressed in combination with the wild-type allele.

**Figure 3:**
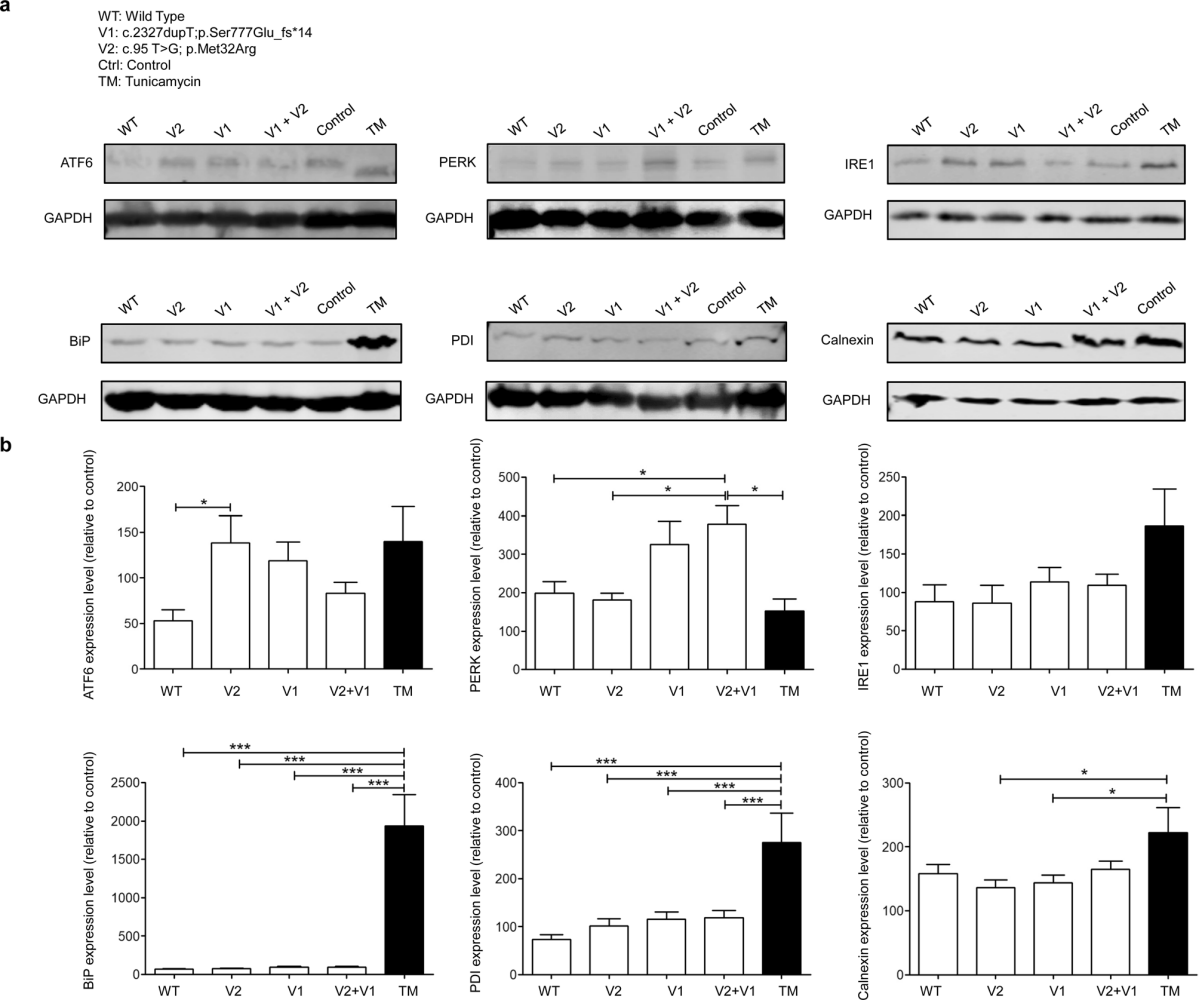
Representative Western blot images and quantifications for ER stress/UPR markers after *COG5* plasmid transfections. (**a**,**b**) Six ER stress/UPR markers were assessed using Western blot analysis including: ATF6 (n = 9), PERK (n = 6), IRE1 (n = 8), BiP (n = 7), PDI (n = 11), and Calnexin (n = 11). (**a**) Representative Western blot images showing protein levels in different in vitro transfection groups: wildtype *COG5* plasmid (WT), (V2) Met32Arg -mutant plasmid, (V1) Ser777Gln_fs*14-mutant plasmid, a co-transfection group of V1 + V2, and a blank insert (control) as a negative control. Cell treated with tunicamycin (TM) were used a positive control. GAPDH staining was used for normalization. (**b**) Quantification of Western blot analyses for the six ER stress/UPR markers. The concentration levels were quantified from n ≥ 6 independent experiments. One-way ANOVA analysis, followed by Tukey post-hoc test (alpha = 0.05) was used to assess levels of statistically significant differences between compared groups. ATF6 (F = 3.262, *p* = 0.0257), PERK (F = 4.759, *p* = 0.0068), IRE1 (F = 2.106, *p* = 0.1038), BiP (F = 35.10, *p* < 0.0001), PDI (F = 9.555, *p* < 0.0001), Calnexin (F = 3.122, *p* = 0.0245) * = *p* < 0.05, *** = *p* < 0.001. Graphs display mean ± S.E.M.

Hypothesizing that disrupting COG5 function could induce ER stress and activation of the UPR, we assessed the levels of several UPR components in vitro*.* N1E-115 cells were transfected with the same amount of plasmid expressing COG5 (WT), V1 ( Ser777Glnfs*14), V2 (Met32Arg) or V1 + V2. A plasmid with a blank insert was used as a negative control, and another group of cells were treated with Tunicamycin (TM) as a positive control as this is known to induce ER stress by blocking *N*-glycosylation. Western blot analysis was used to validate markers of ER stress: calnexin, IRE1, ATF6, PERK, PDI, and BiP. GAPDH served as a housekeeping protein for normalization (Fig. 3 and Supplementary Fig. 4).

IRE1α was the only branch that was not disturbed, with similar protein levels observed in all four *COG5* transfection groups (WT, V1, V2, and V1 + V2). For the second UPR arm, only the V2 transfected group had significantly higher levels of full-length ATF6 compared with the WT (*p* = 0.0257). For the third UPR arm, PERK levels were elevated in cells transfected with both variants (V1 + V2) when compared either to WT, V1, V2 or negative controls (*p* = 0.0068). This suggests that only the combination of the two *COG5* variants (V1 + V2) triggers PERK-protein upregulation, while either variant on its own is not sufficient to promote PERK upregulation. Other ER stress markers BiP, PDI and calnexin were expressed at similar levels across the four *COG5* transfection groups (WT, V1, V2, and V2 + V1).

### Expression of *COG5* mutant constructs resulted in Golgi fragmentation

**Figure 4:**
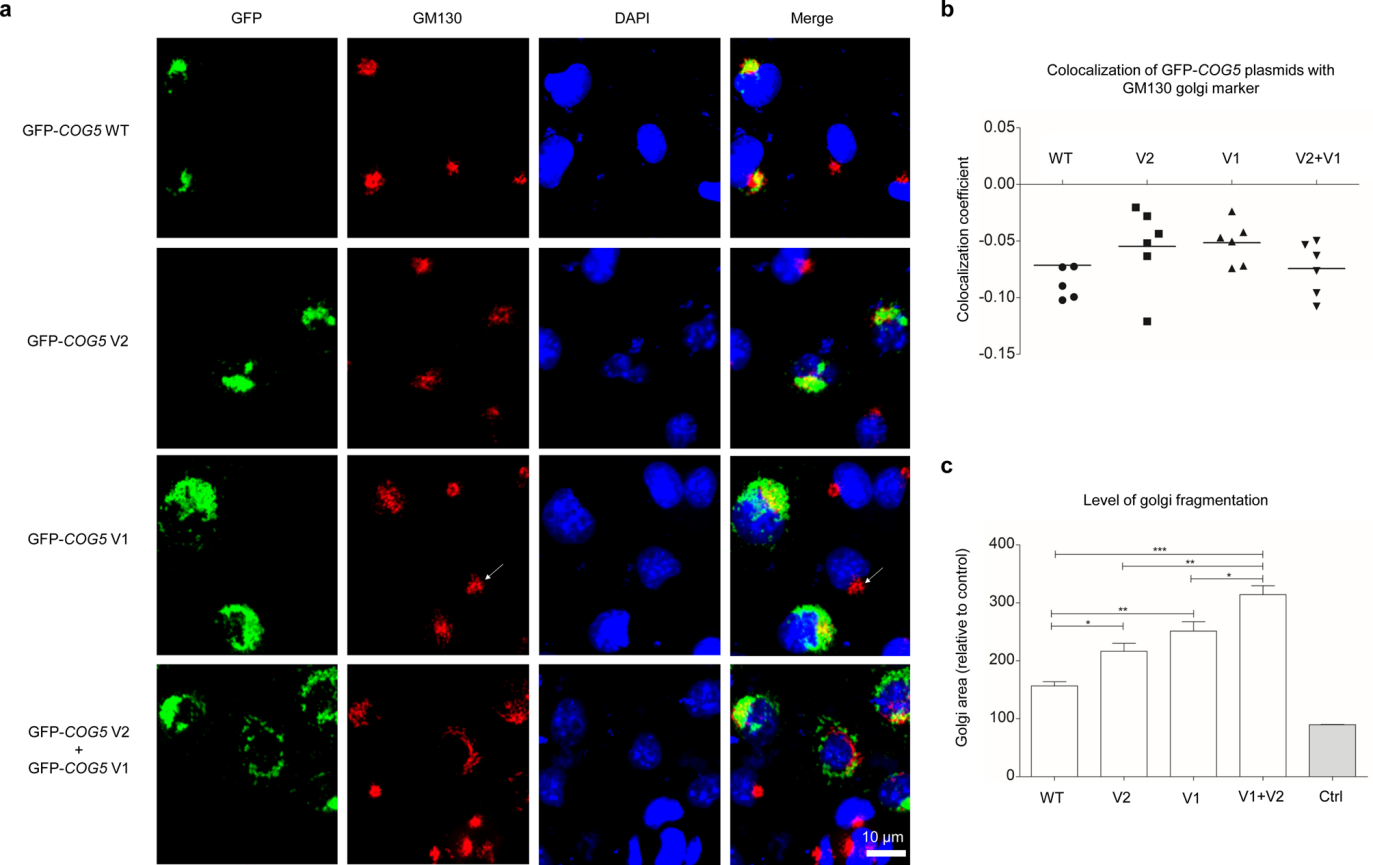
Co-transfection of mutant *COG5* plasmids in vitro leads to increased Golgi fragmentation but no change in plasmid co-localization with Golgi. (**a**) Representative images of immunocytochemical staining performed on N1E-115 cells transfected with GFP-tagged WT, (V1) Ser777Gln_fs*14, (V2) Met32Arg plasmids or co-transfected with V1 + V2 plasmids. GM130 was used as a *cis*-Golgi marker. DAPI was used for nuclear staining. Arrow points towards a normal Golgi structure in an un-transfected cell. Scale bar is 20 μm. (**b**) Quantification of the co-localization between the GFP that is tagged to the *COG5* constructs (WT; V1; V2) and GM130. For each group, > 350 cells were quantified across n = 6 independent experiments. Statistical analysis was performed using one-way ANOVA analysis followed by Tukey post-hoc test (alpha = 0.05; F = 0.8208, *p* = 0.4976). Data presented is mean ± S.E.M. (**c**) Quantification of the relative Golgi area, indicating the level of Golgi dispersal, compared to the control across the four transfection groups (WT; V1; V2; and V1 + V2). For each group, > 150 cells were quantified across n = 3 independent experiments. Results were normalized to the level of Golgi area in the non-transfected control cells. Statistical analysis used one-way ANOVA analysis followed by Tukey post-hoc test (F = 50.42, *p* < 0.0001). * = *p* < 0.05, ** = *p* < 0.01, *** = *p* < 0.001. Data presented is of mean ± S.E.M.

As ER stress often correlates with impaired Golgi trafficking, we studied whether *COG5* variants affected Golgi structure. First, we compared the localization of the WT and mutant (V1, V2 and V1 + V2) COG5-GFP constructs within the cell using the Golgi marker GM130 (Fig. 4a). All COG5-GFP constructs (V1, V2, V1 + V2 and WT) showed similar co-localization of GM130 with GFP (Fig. 4b), however the appearance of the GM130 stained Golgi was different between the four transfection groups. The three mutant COG5-GFP groups (V1, V2, V1 + V2) lead to increased Golgi dispersion when compared to control or WT COG5-GFP (Fig. 4c). The level of Golgi dispersal in the combination transfection group (V1 + V2) was considerably higher than all other groups, indicating that the combination of both variants had additive effects on Golgi dispersion when compared to each variant on their own.

### V1 + V2 transfection induced PERK-mediated DNA damage

**Figure 5:**
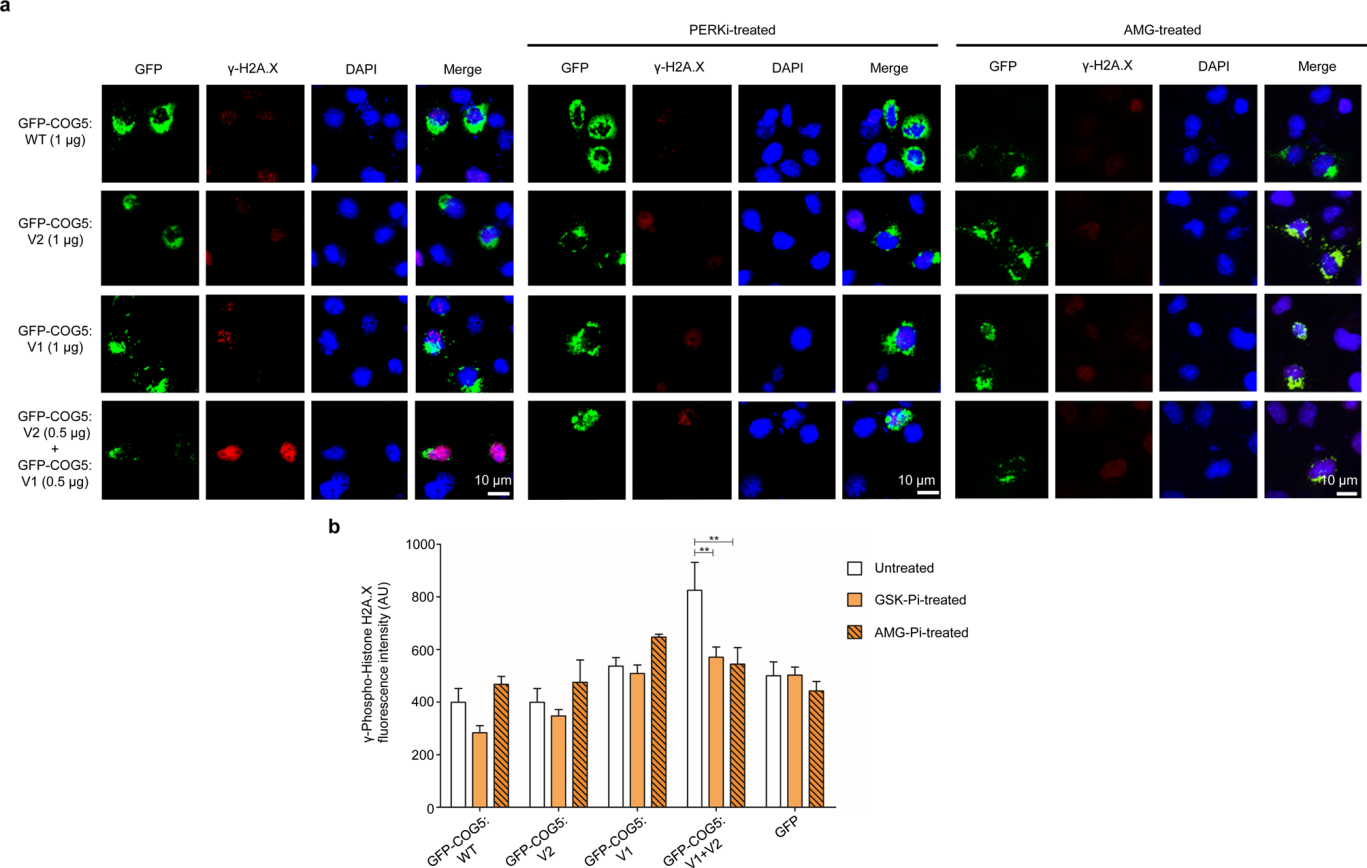
Compound heterozygous *COG5* variants result in increased DNA damage in vitro that is rescued with PERK inhibitor treatment. (**a**) Representative images of N1E-115 cells transfected with wildtype or mutant [(V1) Ser777Gln_fs*14 or (V2) Met32Arg] *COG5* plasmids that are tagged with GFP. The concentration of the DNA plasmid transfected is represented in brackets. Left, untreated cells; Middle, cells treated with GSK2606414 PERK inhibitor; Right, cells treated with AMG PERK 44 inhibitor. The cells were immuno-stained against γ-H2A.X. DAPI was used for nuclear staining. Scale bar is 20 μm. (**b**) Quantification of γ-H2A.X fluorescence intensity, in arbitrary units (AY), measured in the transfected cells as represented in (**a**). For each group, n > 125 cells were measured and pooled from n ≥ 3 independent experiments. Comparison of γ-H2A.X fluorescence intensity between the untreated (n = 6) and PERK inhibitors (GSK [n = 6] or AMG [n = 3])-treated groups in the different *COG5* transfection cells. Statistical significance was assessed using ANOVA analysis followed by Tukey post-hoc test (alpha = 0.05; F = 2.35, *p* = 0.0301). ** = *p* < 0.01. Data presented is of mean ± S.E.M.

Previous studies have demonstrated a clear link between Golgi fragmentation and DNA damage^17^. Hence, we studied the level of DNA damage in each of the four-transfection groups using an anti-gamma-phospho-histone H2A.X antibody (γ-H2A.X), a marker for double-stranded DNA breaks. Transfection of mouse neuroblastoma N1E-115 cells with the combination of V1(Ser777Glnfs*14) + V2 (Met32Arg) showed increased γ-H2A.X staining (Fig. 5a,b).

**Figure 6:**
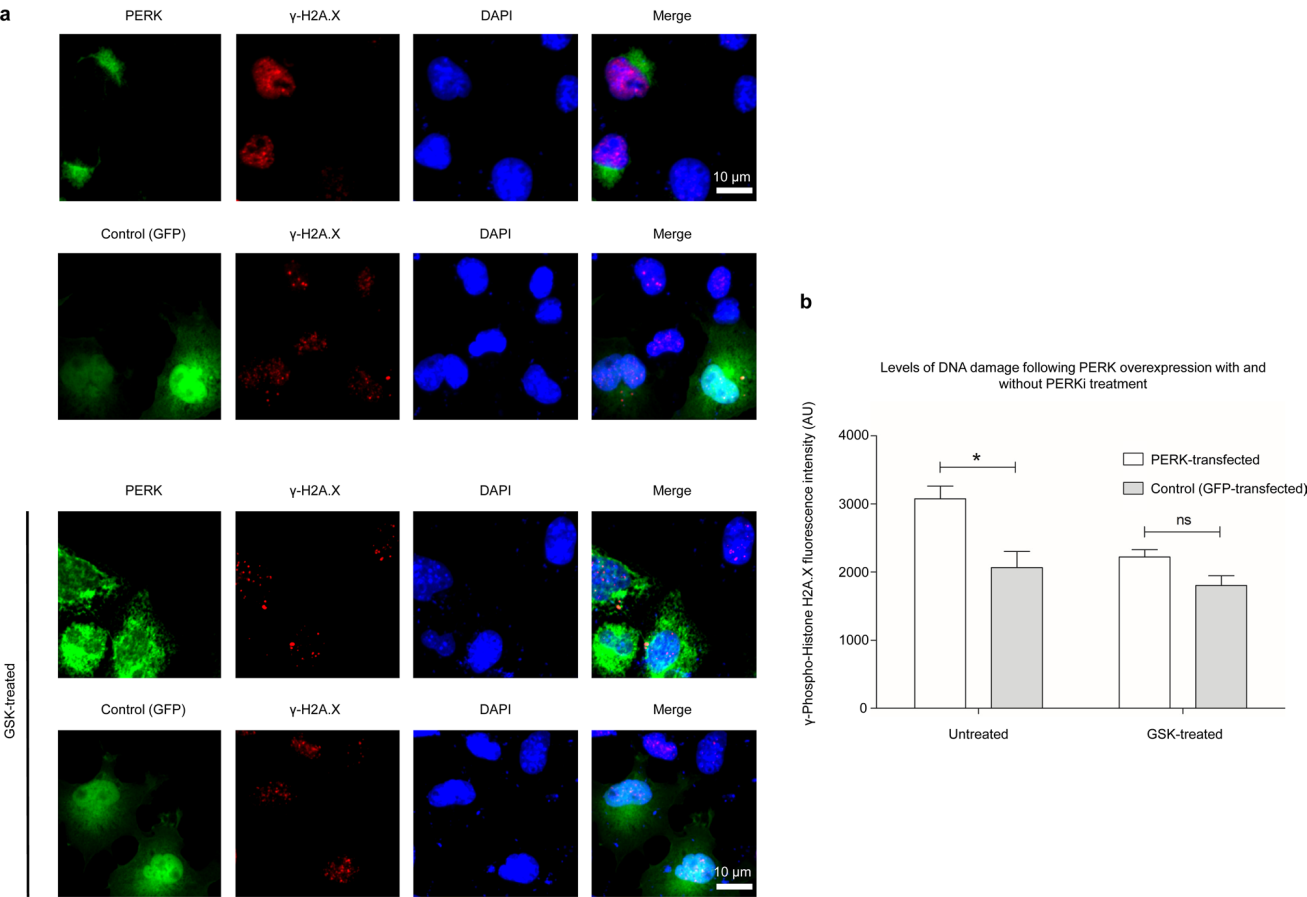
Overexpression of PERK protein leads to increase in DNA damage. (**a**) Representative images of N1E-115 cells that were transfected with either a plasmid expressing PERK or GFP as a control. Top, untreated cells; Bottom, cells treated with GSK2606414 PERK inhibitor (PERKi). Scale bar is 20 μm. (**b**) Quantification of γ-H2A.X fluorescence intensity, in arbitrary units (AU), measured in cells transfected with PERK (n = 3) or GFP (n = 3) and treated (n = 3) or untreated with PERKi (n = 3). For each group, n > 150 cells were measured and pooled from n ≥ 3 independent experiments. Statistical significance was assessed using a two-tailed Student t-test (*p* = 0.0283). * = *p* < 0.05, ns = not significant. Data presented is of mean ± S.E.M.

Given that the V2 + V1 co-transfection cells also had significantly raised PERK protein levels (Fig. 3b), we tested whether PERK up-regulation influenced DNA damage by using PERK inhibitors (PERKi). We treated transfected cells with two PERKi that are not structurally related: AMG PERK 44 (AMG) and GSK2606414. Staining with γ-H2A.X 24 h post-transfections showed that both GSK and AMG treatment significantly reduced the level of γ-H2A.X. PERK upregulation was furthermore shown to be sufficient to causes DNA damage by transfecting N1E-115 cells with a PERK-expressing plasmid. The γ-H2A.X fluorescence intensity was significantly increased following PERK transfection when compared to a GFP control transfection group (Fig. 6), indicating higher levels of DNA damage. Inhibition with GSK2606414 restored DNA damage to control levels (Fig. 6a,b). Together our results suggest that combining the two COG5 variants (V1 + V2) leads to PERK upregulation and subsequent DNA damage, which in turn is detrimental to cell survival.

### PERK is expressed in the retina and overexpression causes photoreceptor death

**Figure 7:**
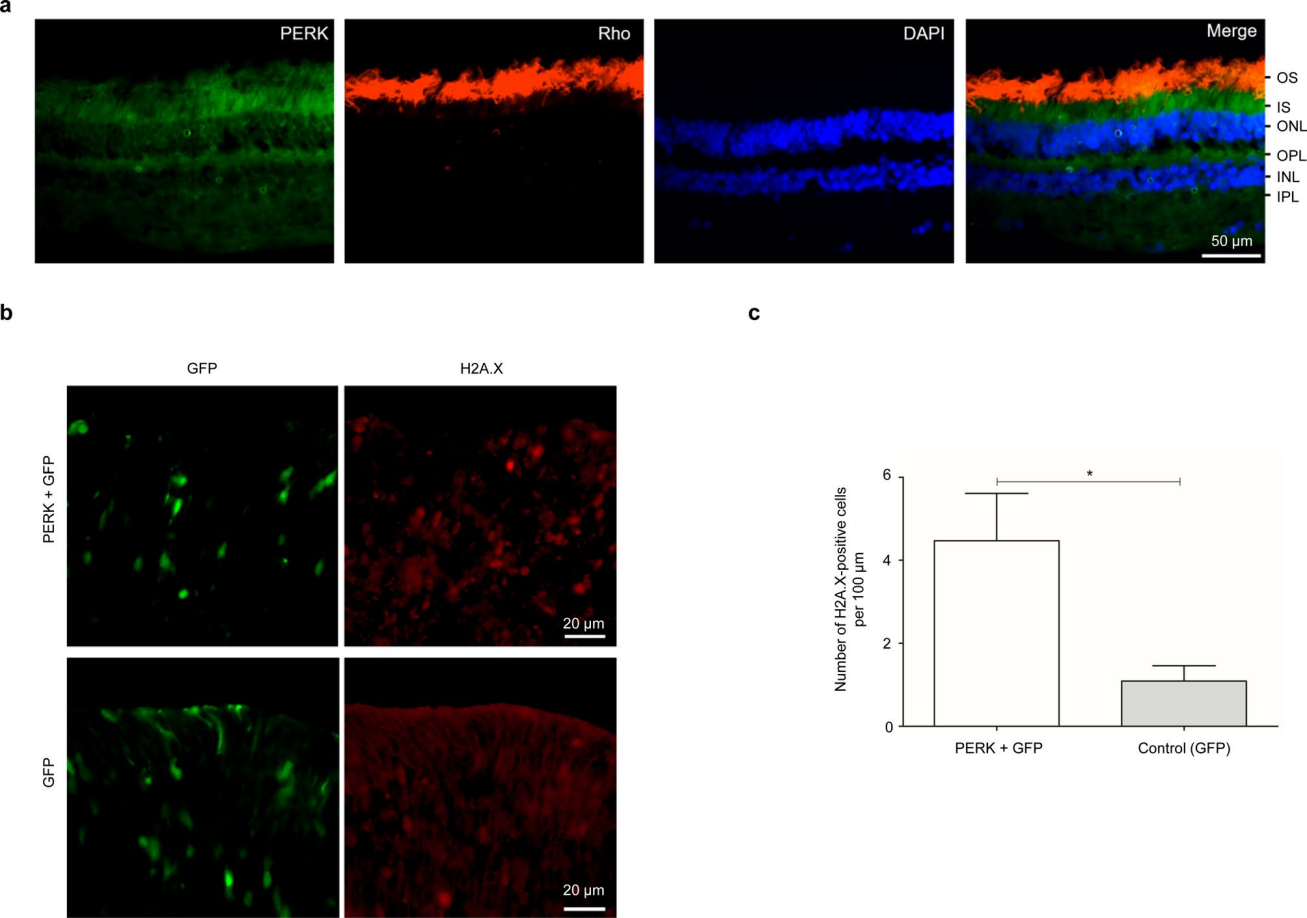
PERK is expressed in the retina and its ectopic expression induces DNA damage. (**a**) PERK is expressed in the inner segments of human photoreceptors. Immunohistochemical staining performed on a sectioned retina from an adult human male. Rhodopsin (Rho) staining marks the outer segments (OS) of the photoreceptors. PERK staining is strongest in the inner segments (IS) of the photoreceptors. DAPI was used for nuclear staining. ONL = Outer nuclear layer; INL = Inner nuclear layer; IPL = Inner plexiform layer. Scale bar is 50 μm. (**b**) PERK over-expression induces DNA damage in photoreceptors. Immunohistochemical staining of γ-H2A.X on mouse retinas that were electroporated with a plasmid expressing PERK (as well as a GFP plasmid for visualization) (n = 3), and control retinas that were electroporated with a GFP plasmid only (n = 3). Scale bar is 20 μm. (**c**) Quantification of the number of H2A.X-positive cells counted within an area of the retina that was visibly transfected with GFP, and normalized to the distance of the transfected area measured. Statistical significance was assessed using a two-tailed Student t-test (*p* = 0.0478). * = *p* < 0.05. Error bars = S.E.M.

Using immunohistochemistry, we observed that PERK, similar to COG5, was expressed in the inner segments of photoreceptors in the human retina (Fig. 7a). Given that we show that PERK upregulation can trigger DNA damage in vitro in NIE-115 cells, we next sought to determine if elevated PERK can similarly induce DNA damage in vivo in mouse photoreceptors. We performed subretinal injection followed by in-vivo electroporation of a plasmid expressing PERK in C57BL/6J P0-P1 mouse retina. Electroporation of the P0 mouse retina transduces dividing cells, leaving post-mitotic cells unaffected. Most of the cells generated from the electroporated newborn progenitors are rod photoreceptors^18^. Two days following electroporation, eyes were collected and γ-H2A.X staining was performed. While electroporation of a control plasmid in the eye generated few γ-H2A.X positive cells, the overexpression of PERK significantly increased DNA damage (Fig. 7b,c). Thus, in agreement with a role of PERK on DNA fragmentation, we showed that PERK upregulation alone is sufficient to cause photoreceptor death in the murine retina.

## Discussion

We identified two *COG5* variants that when compounded act as a novel cause of complex LCA, for which we propose a potential mechanism whereby these variants promote photoreceptor dysfunction. In the human retina, we observed that both PERK and COG5 localize in photoreceptors inner segments and that the COG5 expression is greater in cones than in rods. In-vitro, we showed that combined expression of the two COG5 variants (V1 + V2) leads to Golgi fragmentation and PERK up-regulation. PERK up-regulation in turn was sufficient to induce DNA damage both in human cell cultures and in-vivo in murine photoreceptors. Our study described for the first time novel relationships between *COG5* subunit variants, UPR activation, DNA damage, and subsequent retinal degeneration in humans without neurocognitive impairment. Hence, *COG5* becomes a candidate gene for complex retinal degeneration, especially when short stature and microcephaly are present.

Variants in almost all eight COG subunits have been linked to congenital disorders of glycosylation (CDGs), which include a various disabilities ranging from multi-organ failures, dysmorphism, skeletal malformations to hormonal disorders and coagulopathies, but not retinal degeneration^6,7,16^. In mammalian cells, the 8 subunit COG complex is a Golgi transport complex responsible for retrograde transport of intra-Golgi components. COG5 is part of lobe B of the bi-lobe COG complex, which may act in docking and/or fusion of molecules within the Golgi. The clinical manifestations associated with COG variants depends on the type of glycosylation affected. Specifically, *COG5* variants usually lead to CDG type ii, with a phenotype that generally includes moderate psychomotor retardation, language delay, ataxia, slight hypotonia and microcephaly^6^. Other features such as short stature, cortical blindness and deafness have also been reported, but again not retinal degeneration or LCA^19^. Consequently, these set of genes and related pathways have not been considered as candidates for variant analysis for LCA or IRD, where variants remain unidentified in 30–40% of cases. We suspect that the prevalence of retinal degeneration in CDG could be underestimated. Children may present with nystagmus without retinal changes because of the early stage of disease; without electroretinography the retinal dysfunction could easily be missed. With the advent of subretinal gene therapy recently approved by the FDA^2^, IRDs are becoming actionable diseases, which raises the incentive for identifying disease-causing variants^1,3,20–22^.

We showed that *COG5* variants induced ER responses with upregulation of PERK and activation of UPR. The high metabolic demand of the retina makes it very susceptible to damage from excessive ER stress^23^. Dysregulation of the UPR in response to ER stress has been documented in other photoreceptor diseases such as retinitis pigmentosa and achromatopsia^24,25^. Here we specifically saw elevation in PERK protein levels, however it should be noted that alterations in PERK levels can lead to differential results depending on the cellular and environmental context^26^. Furthermore, ER stress responses can be influenced by aging, genetic variants and environmental factors^26^. Previous data demonstrated that PERK activation is part of a protective response to mutant rhodopsin that ultimately limits photoreceptor cell death^24^, however our data indicates that the overexpression of PERK induces DNA damage which was rescued by PERK inhibition. This argues that PERK’s influence on photoreceptor health is context specific, and indicates that individualized attention to causative molecular pathways related to IRDs are warranted, as targeting the right pathway may lead to new therapeutic opportunities.

Our studies also associated *COG5* variants to Golgi fragmentation. Identifying the mechanisms leading to Golgi fragmentation is beyond the scope of the present study, nevertheless we can speculate on the origin of that association. Fraber-Katz et al. suggested that DNA damage induces Golgi reorganisation/ fragmentation independent of apoptosis^17^. Thus, it is possible that PERK upregulation due to *COG5*^V1/V2^ induces DNA damage which results in Golgi fragmentation. Whether the Golgi fragmentation is caused by PERK upregulation, or the inherent existence of a mutant COG5 subunit, remains unclear.

In conclusion, we identified a direct link between the expression of two *COG5* variants and (1) upregulation of the UPR stress marker PERK, (2) resulting DNA damage and (3) photoreceptor degeneration in human patients. While previous studies have identified integral components downstream of PERK activation that are necessary for γ-H2A.X recruitment and DNA damage^13^, the exact mechanism of how PERK upregulation causes DNA damages remains unknown. The ability to reverse PERK upregulation as shown in vitro offers promising therapeutic avenues. By furthering our understanding of the specific effects of COG5 disruption on cellular function, we may also gain mechanistic insights into how variants in other subunits, such COG7 and COG6, may promote pathogenesis in other disorders^19,27^. Ultimately, the continued identification of novel variants and subsequent investigations into the molecular effects of such variants provides opportunities for the development of novel therapeutics to improve function or limit degeneration.

## Methods

### Patients

This project was approved by the Ethics Review Board of the Hospital for Sick Children (Toronto, Canada) and met the Tenets of the Declaration of Helsinki. Patients were recruited from the Hospital for Sick Children Eye clinic. Consent was obtained from subjects’ parents. Phenotype information was obtained from the medical chart and direct examination. All participants were examined. The ocular phenotype was defined by parameters of visual function (visual acuity, color and contrast vision, visual field, electroretinogram) and of ocular/retinal structure (slit-lamp examination, retinal imaging). DNA from the five family members (Fig. 1a) underwent clinical genetic, which excluded exonic variants in known LCA or IRD-associated genes (data not shown).

### Genotyping, linkage analysis and copy number variation (CNV)

The five participants were first genotyped using the HumanCoreExome-24v1-0_A.bpm microarray chip (*Illumina*) at The Centre for Applied Genomics (TCAG, Sickkids Hospital). The proportion of non-missing genotype (call rate) across the genome per sample was calculated using PLINK v1.90b3.44 (https://www.cog-genomics.org/plink2). The software KING 1.4^28^ was used to compare the observed genotypes with the relationships provided in the pedigree information.

Simulations under the alternative hypothesis of linkage were performed in SLINK 3.0.2^29^ assuming dominant and recessive models, each with penetrance of 50, 60, 70, 80, 90, 95, and 99%. The phenocopy rate was fixed at 0.2% for all models. Three different disease allele frequencies (*q*) were used: 0.001, 0.01 and 0.1 for the dominant model, and 0.05, 0.15 and 0.3 for the recessive model, corresponding approximately to disease prevalence of 0.2, 2 and 10%, respectively, for fully penetrant models. Each model and parameters were analyzed using Merlin 1.1.2^30^. The maximum LOD score obtained from the analysis of 500 simulated pedigrees was declared the maximum LOD score for any particular model. Before linkage analysis, monomorphic markers (SNPs) were removed within the five individuals, as well as markers not in the autosomal chromosomes, and the ones ambiguous for strand information. Additionally, to avoid inflation of multipoint LOD scores, the marker set was filtered to remove linkage disequilibrium (LD) using data from samples of East Asian population from the 1000 Genomes Project^31^ and from microarray data^32^. CNV was inferred by the Genotyping data using GenomeStudio (*Illumina*) with the plugin CNVpartition v3.2.0 (*Illumina*) for the 5 family members.

### Genome sequencing and variant analysis

Pair-end (2 × 150 base pairs) Genome Sequencing (GS) was performed on affected individuals II-1 and II-3 using Illlumina Hi-Seq X with mean coverage of 39.4 times. The sequence filtering pipeline used is Summarize in Supplementary Fig. 1. Reads were mapped to the human b37 reference sequence using bwa-mem v0.7.12^33^. Duplicate reads were removed using MarkDuplicates from Picard v2.5.0 (https://broadinstitute.github.io/picard/). Local read realignment around indels, base quality score recalibration (BQSR), variant calling with HaplotypeCaller, and variant quality score recalibration (VQSR) were accomplished using GATK v3.7.0^34^. Variant calls were annotated using a custom pipeline developed at The Centre for Applied Genomics (TCAG) at SickKids based on ANNOVAR^35^. Mobile element insertion detection was performed with Mobster v0.2.4.1^36^ adjusting parameters for minimum clip read length of 15 nucleotides and 3 minimum support reads. Linkage results were used a posteriori to prioritize variant segregation. GnomAD^37^ control population database for genomic data was used at the end to further eliminate variants, as that newer version of the database was not included in the original annotation.

### Protein model generation

The sequence for COG5 was obtained from Uniprot (https://www.uniprot.org) and fed into the Phyre2 server to generate the presented homology model^38^. The top model that encompassed the amino acid changes induced by the V1: c.2327 dupT alteration (C-terminal half of the protein) possessed a model confidence score of 98.3%, using the sec6 experimental structure as a template (PDB ID 2FJI). A structural model that included the amino acid residues altered as a result of the frameshift was generated similarly. These models were used for all analyses presented. Figures were produced with Pymol^39^.

### Mice

C57BL/6 J mice were purchased from Jackson Laboratory and handled in accordance with the ethical and legal requirements outlined by Ontario’s Animals for Research Act and the federal Canadian Council on Animal Care. All experiments performed were approved by the University Health Network Research Ethics Board as overseen by the Animal Resources Centre at the Krembil Research Institute.

### DNA constructs and mutagenesis

Construct of human *COG5* (cDNA clone MGC: 87,389 IMAGE: 4,374,289) in a pcDNA 6.2 vector and tagged at the N-terminus with GFP was obtained from The SickKids Proteomics, Analytics, Robotics & Chemical Biology Centre (SPARC BioCentre). PERK 1: PERK.WT.9E10.pcDNA was a gift from David Ron (Addgene plasmid # 21,814). Site-directed mutagenesis was performed on the *COG5* plasmids to insert the variants identified: V1: p.Ser777Gln_fs*14 and V2: Met32Arg. This was done by preparing a mutant strand synthesis reaction mixture of 10 µl 5X GC reaction buffer, 100 ng of dsDNA template, 2.5 µl of 10 µM forward primer, 2.5 µl of 10 µM reverse primer, 1 µl of 10 mM dNTP, 1.5 µl of DMSO, 0.5 µl of Phusion DNA polymerase (NEB), and up to 50 µl of nuclease-free, sterile, double-distilled water (ddH_2_O). Polymerase chain reaction (PCR) cycle consisted of: (1) 1 cycle of 95 °C for 30 s; (2) 18 cycles of (a) 98 °C for 30 s, (b) 60 °C for 1 min, (c) 68 °C for 1 min per kilobase of plasmid length. At the end of the reaction, samples were placed on hold at 4 °C. The parental DNA was digested by adding 1 µl of *Dpn* I restriction enzyme (NEB) to the 50 µl reaction mixture and incubated at 37 °C for 1 h. DNA was transformed into competent DH5ɑ *Escherichia coli* (*E. coli*) cells and plated on LB-agar plates. For Western blotting applications, the GFP was excised out and replaced with a 6xHistadine tag. This was performed by creating primers that include a restriction enzyme as well as the 6xHistadine sequence and running a PCR to amplify the gene insert attached to the 6xHis tag. The PCR product was run on an agarose gel and extracted using QIAquick Gel Extraction Kit (Qiagen), polyadenylated, and then purified using the QIAquick PCR Purification Kit (Qiagen). The DNA was ligated into pGEM®-T Easy Vector for white-blue selection of colonies. Selected positive colonies were inoculated for plasmid preparation and DNA sequencing was confirmed. The DNA insert was digested out of the T Easy Vector and ligated into an empty pcDNA 6.2 vector. Verification of all mutants and constructs was done through sequencing of DNA at ACGT Corporation. DNA plasmid preparation and purification were performed using the Plasmid Mini and Max Kits (Qiagen), according to the manufacturer’s manual.

### In-vivo electroporation

Electroporations of P0 mouse retina were performed as previously described^18,40^. Briefly, maxi preps of the plasmids were prepared (Qiagen) and were diluted to working concentrations of 1 µg/µl. 0.1% fast green dye was added to DNA solutions prior to injection. Subretinal injections (volume approximately 0.3 µl) were performed using a 10 µl Hamilton syringe coupled with a blunt end 0.5″ 32 gauge needle tip. Five pulses pulse of 80 V for 50 ms followed by a 950 ms interstimulus interval were administered using 10 mM tweezer electrodes.

### Cell culturing, transfection, and lysis

N1E-115 (mouse neuroblastoma) and HEK-293 (human embryonic kidney) cells were cultured in DMEM (Sigma-Aldrich) supplemented with 10% FBS (Gibco) and 1% Penicillin–Streptomycin (Gibco). Patient-derived lymphoblasts were cultured in RPMI-1640 supplemented with 15% FBS. N1E-115 cells were used due to being morphologically similar to neuronal cells in the retina, while HEK-293 cells were used for increased transfection efficiency for assessing global changes to ER stress and UPR markers in cell lysates through Western blotting. Lipofectamine 3000 reagent (ThermoFisher Scientific) was used for N1E-115 transfection in 10 cm plates according to the manufacturer’s manual. Polyethyleneimine (PEI) was used for HEK-293 transfection by mixing 9 µg of DNA with 27 µl of PEI in 500 µl of Opti-MEM. The transfection mixture was incubated at room temperature for 15 min prior to adding it on top of the supplemented growth media for cultured cells. The media was replaced with fresh supplemented media 6–16 h after transfection. Transfected cells were fixed or lysed 24–48 h after transfection, as per experimental objectives. Cell lysis was performed using 1X RIPA lysis buffer (Cell Signaling Technology) with 1X protease inhibitor cocktail (Sigma-Aldrich) as per manufacturer’s instructions. Total protein concentrations in cell lysates were measured using the Pierce BCA Protein Assay Kit (ThermoFisher Scientific).

### Immunocytochemistry and immunohistochemistry

Cells to be fixed for immunocytochemistry were cultured on adhesive round glass coverslips coated with 0.01% poly-L-lysine (PLL) solution (Sigma-Aldrich). After culturing, treatment, and/or transfection, cells were fixed using 4% paraformaldehyde (PFA) for 10 min on ice. After thorough washing, cells were permeabilized in PBS + 0.1% Triton™ X-100 at room temperature for 10 min. Cells were washed and blocked for 30 min at room temperature with 10% FBS (in PBS-T). Cells were then incubated with the primary antibody diluted in blocking buffer overnight at 4 °C. After thorough washing, cells were incubated for 1 h at room temperature with the secondary antibody diluted in blocking buffer. The cells were washed, counter-stained with DAPI, and mounted with Mowiol mounting medium. Primary antibodies used for immunofluorescence include polyclonal rabbit anti-giantin (Biolegend, 19243; 1:4000), polyclonal rabbit anti-GM130 (Cell Signaling Technology, 12480S; 1:1000), and monoclonal mouse anti-phospho-histone H2A.X (Millipore-Sigma, 05–636; 1:1500). Secondary antibodies used are Alexa Fluor Donkey anti-Rabbit 555 and Alexa Fluor Donkey anti-Mouse 555 at 1:500 dilution (Life Technologies).

### Western blot

Cell lysates were mixed with Laemmli buffer plus dithiothreitol (DTT), heated for 5 min at 95 °C, and loaded onto SDS-PAGE (sodium dodecyl sulfate—polyacrylamide gel electrophoresis) gels. The gels were run in Tris–Glycine SDS running buffer and then transferred onto nitrocellulose membranes. Blocking consisted of at 5% skim milk or 2.5% BSA, depending on the primary antibody used. Imaging was performed using the Li-Cor Odyssey fluorescence imaging system. Primary antibodies consisted of: monoclonal mouse anti-6xHis-Tag (Invitrogen, MA1-21315; 1:4000), polyclonal rabbit anti-ATF6 (Novus Biologicals, NBP1-75478; 1:1000), monoclonal rabbit anti-BiP (Cell Signaling Technology, C50B12; 1:1000), monoclonal rabbit anti-Calnexin (Cell Signaling Technology, C5C9; 1:1000), monoclonal rabbit anti-COG5 (Sigma-Aldrich, SAB4200440; 1:600), monoclonal mouse anti-GAPDH (Invitrogen, ZG003; 1:4000), monoclonal mouse anti-GFP (Cell Signaling Technology, 2955S; 1:1000), polyclonal rabbit anti-IRE1a (Cell Signaling Technology, 3294S; 1:1000), monoclonal rabbit anti-PDI (Cell Signaling Technology, 2446S; 1:1000), and monoclonal rabbit anti-PERK (Cell Signaling Technology, D11A8; 1:1000). Secondary antibodies consisted of: Donkey anti-Rabbit IRDye 680, Donkey anti-Rabbit IRDye 800, Donkey anti-Mouse IRDye 680, and Donkey anti-Mouse IRDye 800 (Li-Cor; 1:20,000).

### Image acquisition, analyses, and quantification

Imaging acquisition of the retina and cells used an Olympus BX61 epifluorescence microscope. Images for DNA damage and colocalization analysis were analyzed and processed using CellSens Dimension software. A region of interest (ROI) was drawn around each transfected cell and a scatterplot was generated between the intensity of the two fluorophore wavelengths to be assessed for colocalization. The background levels were subtracted from the scatterplot area of measurement and a colocalization coefficient was generated. The colocalization coefficient was transformed to a Z factor for averaging and the averaged total was transformed back to the R^2^ colocalization coefficient. Levels of DNA damage in cells were quantified by measuring the fluorescence intensity within a drawn ROI around each cell for γH2A.X antibody staining of double-stranded DNA breaks. Exposure time and gain were kept constant among all measured samples, and background fluorescence was subtracted from every image taken. Western blot imaging used the Li-Cor Odyssey fluorescence scanner and Li-Cor’s Imaging Studio was used for Western blot analysis and processing. Protein band intensity measurement and subtraction of background intensity were performed as per manufacturer’s instructions. The protein band intensity was normalized by measuring the intensity of GAPDH bands.

### Statistical analysis

Analysis was performed using Microsoft Excel and GraphPad Prism. Statistical significance for comparing multiple groups was determined by applying one-way ANOVA analysis followed by Tukey post-hoc test if a statistically significant difference was found. Comparison between two groups was performed by using a two-tailed Student’s t-test.

### Details of the genetic analysis results—genotyping and linkage

Out of the original 547,644 SNPs on the microarray chip, 535,996 (97.9%) passed the quality filters. All samples passed QC filters for call rate (removed if < 97%) and autosomal heterozygosity. The provided relationships between the five individuals in the family were supported by the genotypes. The two founders were inferred to have East Asian origin, which is consistent with their Filipino ancestry, and there was no evidence of consanguinity. 13,341 SNPs were suitable for linkage analysis after the quality and LD filters.

## Supplementary information

Supplementary information.

Supplementary information.
