# Supplementary material for: COG5 variants lead to complex early onset retinal degeneration, upregulation of PERK and DNA damage
Source: Sci Rep. 2020 Dec 4;10:21269. doi: 10.1038/s41598-020-77394-3 (PMC7718911; doi:10.1038/s41598-020-77394-3)

COG5 variants lead to complex early-onset retinal degeneration, upregulation of PERK and DNA damage

Sami Tabbarah, Erika Tavares, Jason Charish, Ajoy Vincent, Andrew Paterson, Matteo Di Scipio, Yue Yin, Roberto Mendoza-Londono, Jason Maynes, Elise Heon, Philippe Monnier

Supplementary Information

Supplementary Tables and Figures

Supplementary Figure 1. Summary of ERG and visual field testing.

Left, full-field electroretinography (ERG) results from all three siblings and a control under different light conditions (DA - dark adaptation, LA - light adaptation) with different intensity of flash in cd.s.m-2). All three siblings had cone-rod dystrophy of varying severity; with no recordable cone function. For an unclear reason the eldest affected (Case 1, 21 yrs) retained the most residual rod function whereas the youngest sibling (Case 3, 18 yrs) had the least. Dark adaptation (rod) traces are to the left and light adapted recording (cones) are to the right. The numbers next to DA and LA is the stimulus intensity used. The first negative wave (a wave) is the photoreceptor response while the second wave (positive), the b wave, is the second order neuron response. Y axis of graphs: amplitude in microvolts. X axis of graphs latency of response after stimulus in milliseconds.

Right, are the corresponding visual field tracings (blue line). The blue line traces the only area of the field straight ahead that the patient can see. The center of the circled field is not shade (scotoma) due to the central macular changes. There is loss of peripheral and central sight. Dotted grey line outlines a normal visual field. The gray dotted lines mimics a normal visual field diameter with a clear center.

Supplementary Figure 2.

Cog5 is is preferentially expressed in cones over rods inner segments.

(a) Left: Immunohistochemical staining performed on a sectioned retina from an adult human male showing COG5 (green) as seen in Figure 1. Dashed box represents magnification seen on right. Right: contrast adjusted magnification demonstrating COG5 expression in cone inner segments (arrow) and rod inner segments (arrow head). **(b)** Donkey anti-Rabbit Alexa Fluor 488 was used as a negative control for the rabbit anti-COG5 and rabbit anti-PERK staining performed in Figures 1 and 7, respectively. Donkey anti-Mouse Alexa Fluor 55 was used as a negative control for the mouse anti-rhodopsin (RET-P1) staining performed in Figures 1 and 7. DAPI was used as a nuclear stain. Scale bar is 50μm.

Supplementary Figure 3. Summary of the Filtration pipeline used for Genome Sequencing

Supplementary Figure 4. Western Blotting analysis of COG5 and COG5 mutants.

HEK293 cells were transfected with constructs expressing COG5 and its mutants, and Western Blotting analysis was performed two days after transfection. GAPDH was used as a loading Marker. Ctrl, cells transfected with empty plasmid.

Supplementary Tables

Supplementary Table 1. Summary of Linkage Analysis focused on boundaries of the regions with maximum LOD of 1.19.

Supplementary Table 2. Rare variants from GS shared by individuals II-1 and II-3 after prioritization pipeline.

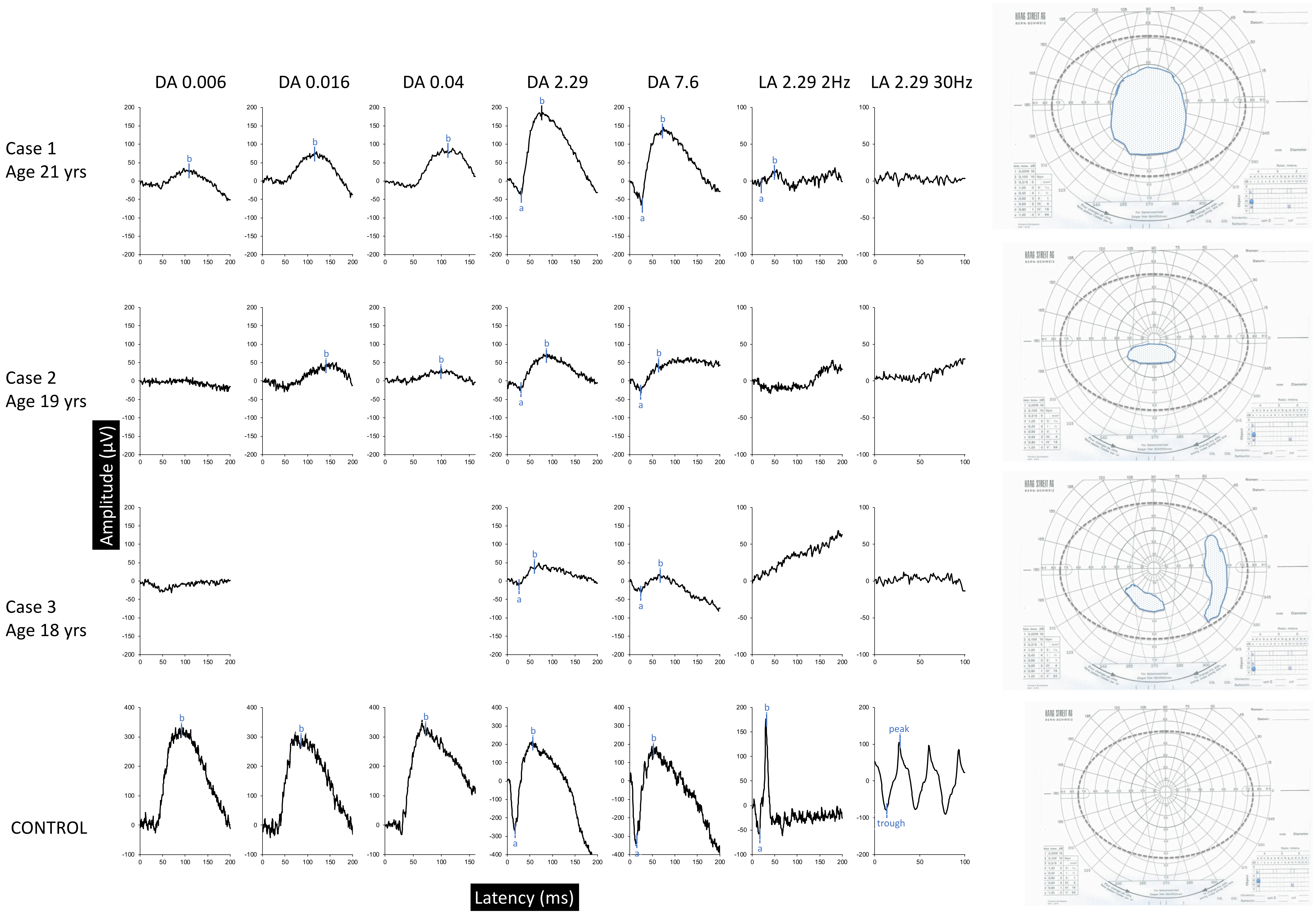
DA 0.006
DA 0.016
DA 0.04
DA 2.29
DA 7.6
LA 2.29 2Hz
LA 2.29 30Hz
Case 1
Age 21 yrs
Case 2
Age 19 yrs
Case 3
Age 18 yrs
CONTROL
Amplitude (µV)
Latency (ms)
a
b
peak
trough
HAAG STREIT AG
BERN-SCHWEIZ

Supplementary Figure 2

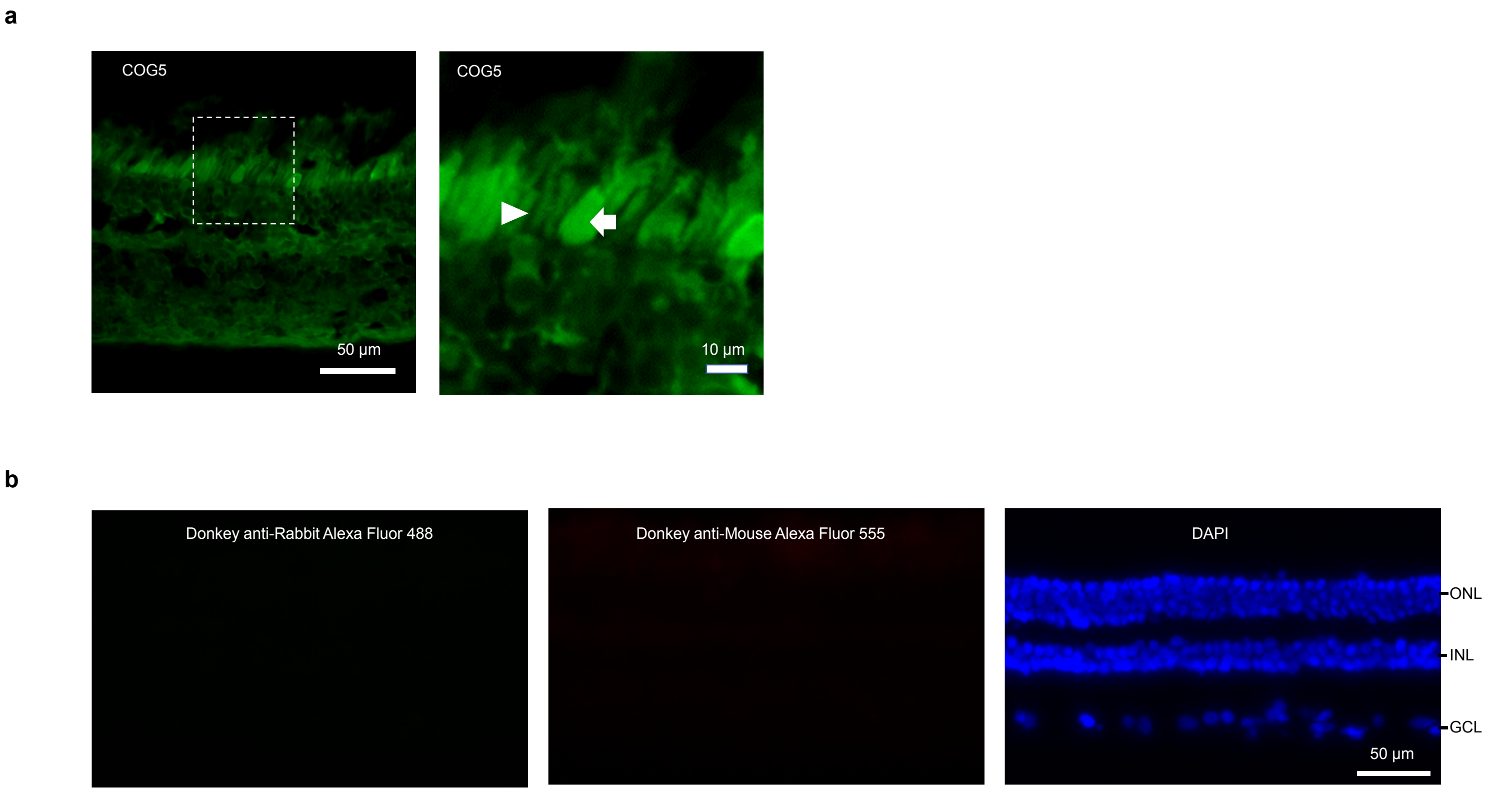

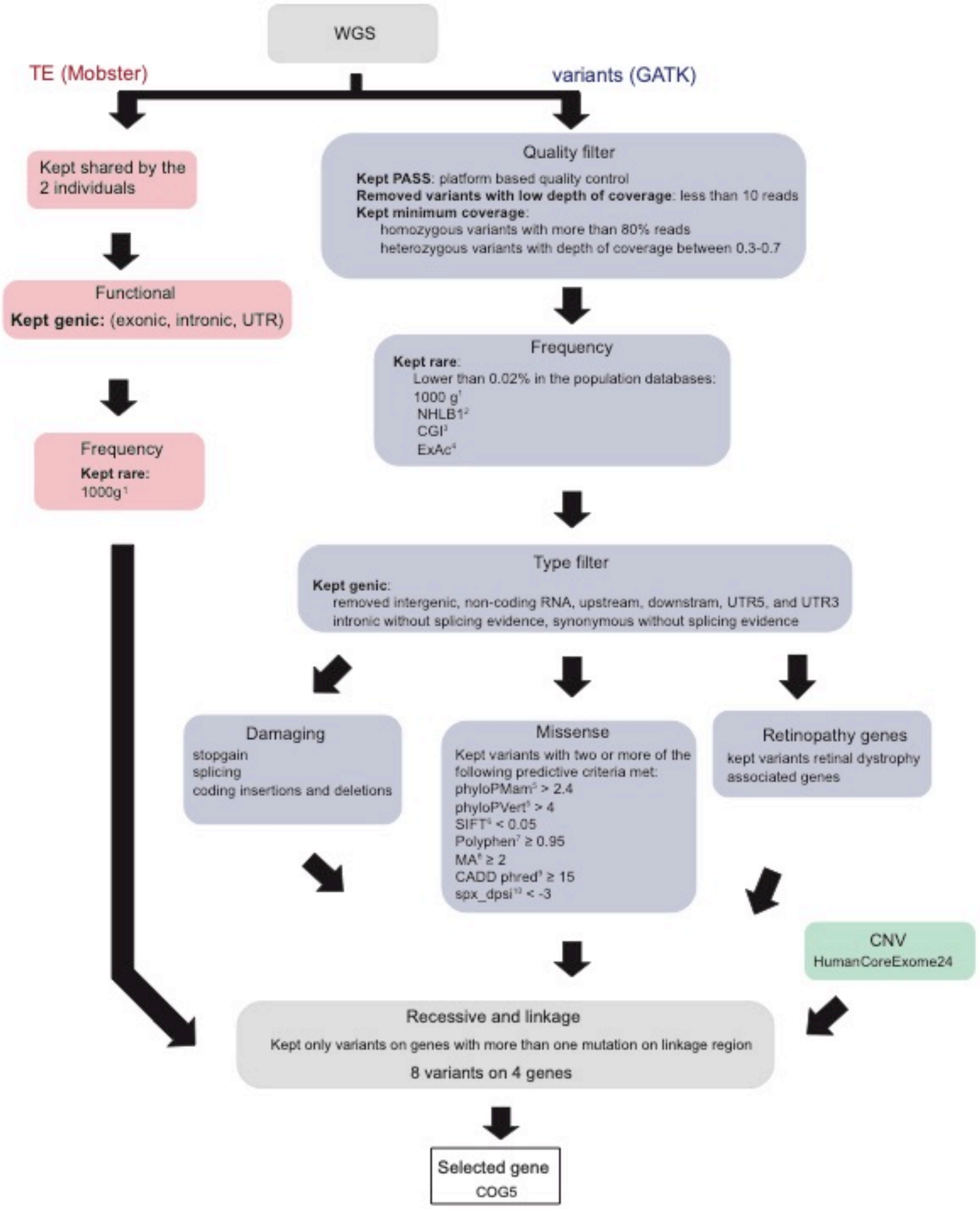

WGS
TE (Mobster)
variants (GATK)
Kept shared by the 2 individuals
Functional
Kept genic: (exonic, intronic, UTR)
Frequency
Kept rare:
1000g[1]
Quality filter
Kept PASS: platform based quality control
Removed variants with low depth of coverage: less than 10 reads
Kept minimum coverage:
homozygous variants with more than 80% reads
heterozygous variants with depth of coverage between 0.3-0.7
Frequency
Kept rare:
Lower than 0.02% in the population databases:
1000 g[1]
NHLB1[2]
CGI[3]
ExAc[4]
Type filter
Kept genic:
removed intergenic, non-coding RNA, upstream, downstram, UTR5, and UTR3
intronic without splicing evidence, synonymous without splicing evidence
Damaging
stopgain
splicing
coding insertions and deletions
Missense
Kept variants with two or more of the following predictive criteria met:
phyloPMam[5] > 2.4
phyloPVert[5] > 4
SIFT[6] < 0.05
Polyphen[7] ≥ 0.95
MA[8] ≥ 2
CADD phred[9] ≥ 15
spx_dpsi[10] < -3
Retinopathy genes
kept variants retinal dystrophy associated genes
CNV
HumanCoreExome24
Recessive and linkage
Kept only variants on genes with more than one mutation on linkage region
8 variants on 4 genes
Selected gene
COG5

Supplementary Figure 4

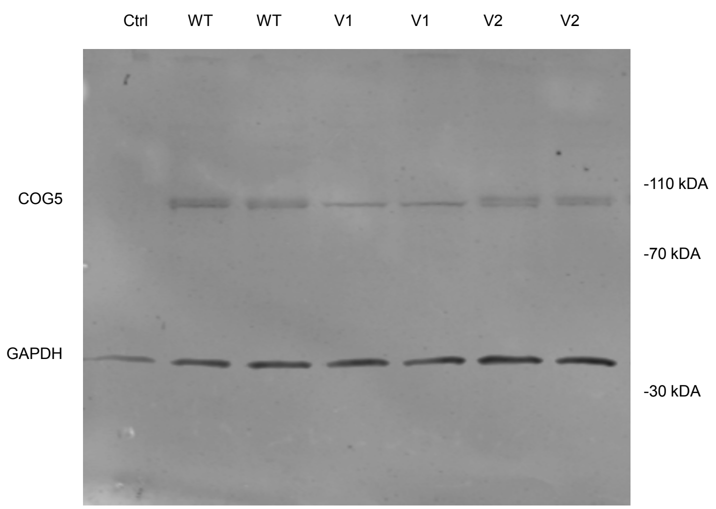

Table S1. Summary of Linkage Analysis focused on boundaries of the regions with maximum LOD of 1.19.

chr	Start		End	
	GRCh37	SNP	GRCh37	SNP
2	119,194,992	rs1370512	129,725,930	rs7601699
2	234,917,732	rs10490018	235,834,973	rs4663523
3	11,896,347	rs303850	40,249,244	rs1344189
7	105,586,853	rs193841	120,145,310	rs802355
8	64,460,952	rs7832904	75,682,535	rs1392966
9	103,947,810	exm767548	109,953,472	rs1464250
9	138,174,978	rs1806807	141,025,328	rs11137376*
10	1,396,155	rs2892333	13,884,259	rs7073646
10	36,862,892	rs1332770	43,448,012	rs3004255
11	12,559,202	rs4757597	19,735,396	exm895707
12	63,225,894	variant.25774	64,976,049	exm2251127
14	56,310,028	rs17128859	69,328,441	rs393673
17	8,547	rs2396789*	1,095,140	rs4417598
19	249,357	kgp12221412*	4,353,024	exm1408874
20	55,772,683	rs230191	56,179,422	rs6064572

Regions boundaries were defined by the 1-LOD support interval. *Marker is on the first or last analyzed SNP on the chromosome, the true boundary is not known. Chr: chromosome, GRCh37: genome map position, SNP: single nucleotide polymorphism. *GRCh37* Genome Reference Consortium Human Build 37 (*GRCh37*) Organism: Homo sapiens (human)

Table S2. Rare variants from GS shared by individuals II-1 and II-3 after prioritization pipeline.

Gene	Genomic vcf chr:position:ref:alt (hg19)	Zygosity	Effect	gnomAD frequency/ # homozygotes	Polyphen	CADD_Phred	Segregation with the disease
TTN	2:179403525:A:T	het	NM_001256850:c.T94108A:p.S31370T	0.01174/ 1	0.027	15.64	No
	2:179447801:A:G	het	NM_001256850:c.T60806C:p.I20269T	0.00229/ 1	0.008	15.1	No
	2:179595849:C:T	het	NM_001256850:c.G16592A:p.G5531E	0.0001927/ 1	1	13.44	No
TTC21A	3:39166625:G:A	het	NM_145755:c.G1189A:p.V397M	0.0007334/ 2	0.912	21	Yes
	3:39178879:G:T	het	NM_145755:c.3471+1G>T (spl)[a]	not seen	NA	10.49	Yes
HTR3E	3:183823729:G:T	homo	NM_198313:c.G852T:p.L284F	0.0006364/0	0.489	12.53	No
COG5	7:106851604:G:GA	het	NM_006348:c.2327dupT:p.F777fs*14	not seen	NA		Yes
	7:107204340:A:C	het	NM_006348:c.T95G; p.Met32Arg[b]	0.0000121/ 0	0.962	18.51	Yes

[a] Splicing predicted (canonical splicing site), loss of natural donor site c.3471, [b] Reported pathogenic for congenital disorder of glycosylation (HGMD # CM1211718,(Rymen et al., 2012)).

Uncropped blots

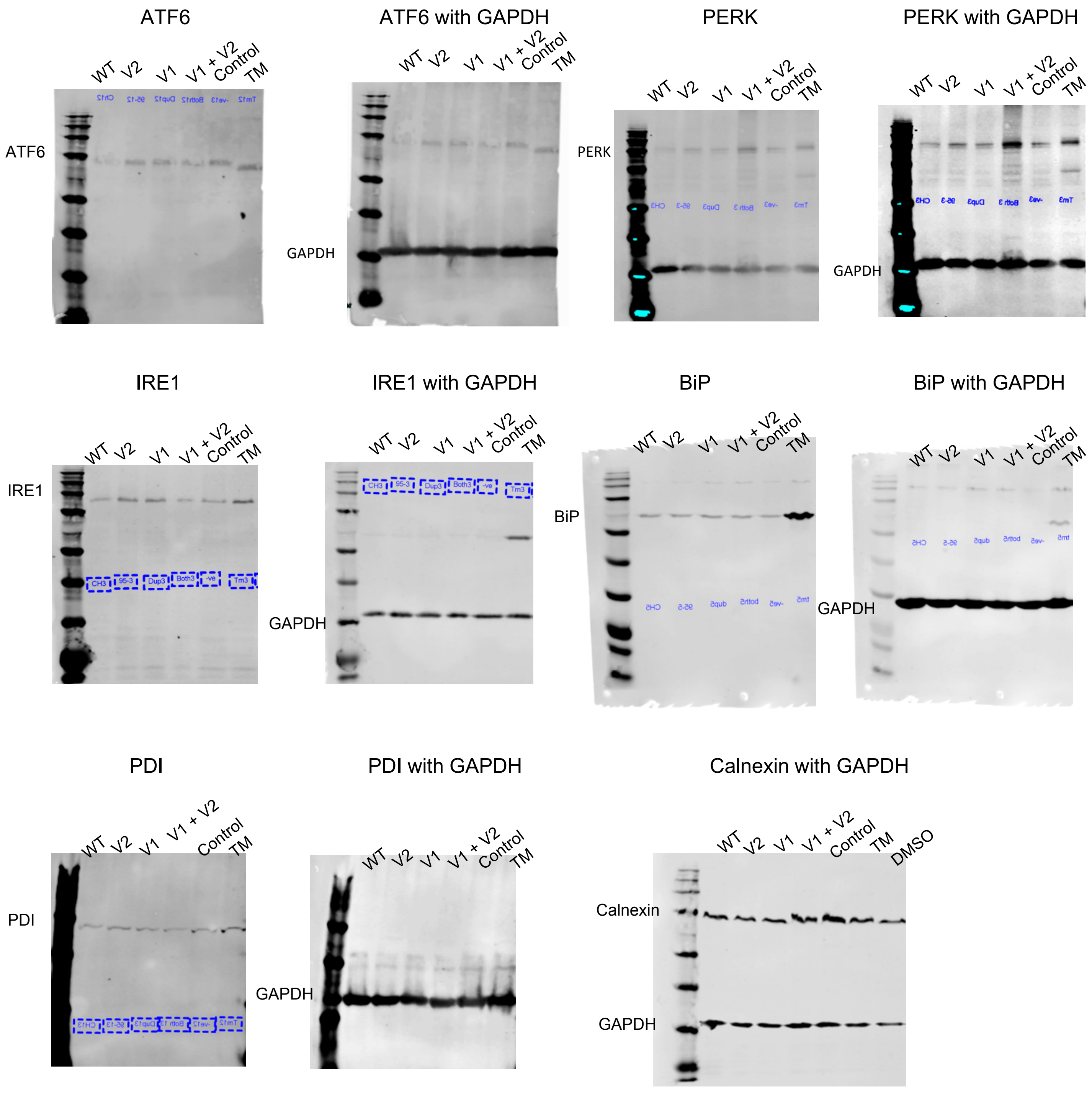

Supplement: Supplementary file 1 — Supplementary information. [file 41598_2020_77394_MOESM1_ESM.pdf]
