# Supplementary material for: COG5 variants lead to complex early onset retinal degeneration, upregulation of PERK and DNA damage
Source: Sci Rep. 2020 Dec 4;10:21269. doi: 10.1038/s41598-020-77394-3 (PMC7718911; doi:10.1038/s41598-020-77394-3)
Supplement: Supplementary file 2 — Supplementary information. [file 41598_2020_77394_MOESM2_ESM.docx]

**Supplementary Material**

**Supplementary Table 1.** Summary of Linkage Analysis

**Supplementary Table 2.** Residual variants from filtering the genome sequences

**Supplementary Figure S1.** Summary of ERG and visual field testing

**Supplementary Figure S2.** Cog5 is preferentially expressed in cones over rods inner segments

**Supplementary Figure S3.** Summary of the Filtration pipeline used for Genome Sequencing

**Supplementary Figure S4.** Western Blotting analysis of COG5 and COG5 mutants
